# Supplementary material for: Improved Survival in Hepatocellular Carcinoma Patients with Cardiac Arrhythmia by Amiodarone Treatment through Autophagy
Source: Int J Mol Sci. 2019 Aug 15;20(16):3978. doi: 10.3390/ijms20163978 (PMC6720201; doi:10.3390/ijms20163978)
Supplement: Supplementary file 1 [file ijms-20-03978-s001.pdf]

Supplementary Table 1 Cox model with hazard ratios and 95% confidence intervals of mortality associated with or without amiodarone treatment among arrhythmia patients without HCC

**Variable**	**Death**	**Crude**			**Adjusted‡ - model 1**			**Adjusted†-model 2**		
	**No.**	**HR**	**95% C.I.**	**p-value**	**HR**	**95% C.I.**	**p-value**	**HR**	**95% C.I.**	**p-value**
**Arrhythmia patients without HCC**										
without Amiodarone	10683	1	reference		1	reference		1	reference	
with Amiodarone	11932	1.15	(1.12-1.17)	0.01	1.13	(0.97-1.26)	0.08	1.05	(0.90-1.34)	0.10

Model 1: Adjusted HR‡ represented adjusted hazard ratio: mutually adjusted for amiodarone drug used, age, gender, HBV, HCV, and liver cirrhosis in Cox proportional hazard regression.

Model 2: Adjusted HR† represented adjusted hazard ratio: mutually adjusted for amiodarone drug used, age, gender, HBV, HCV, HE, ascites, EGV, and treatment in Cox proportional hazard regression.

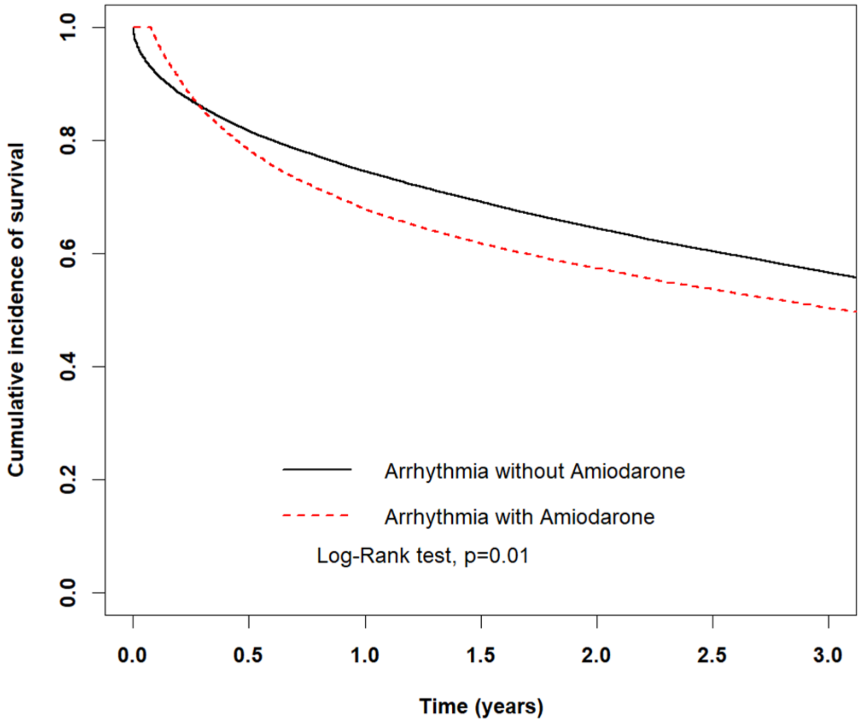

Supplementary Figure 1 Cumulative incidence of survival in arrhythmia patients without hepatoma at the presence and absence of amiodarone by Kaplan-Meier method analysis for 3 years follow up.

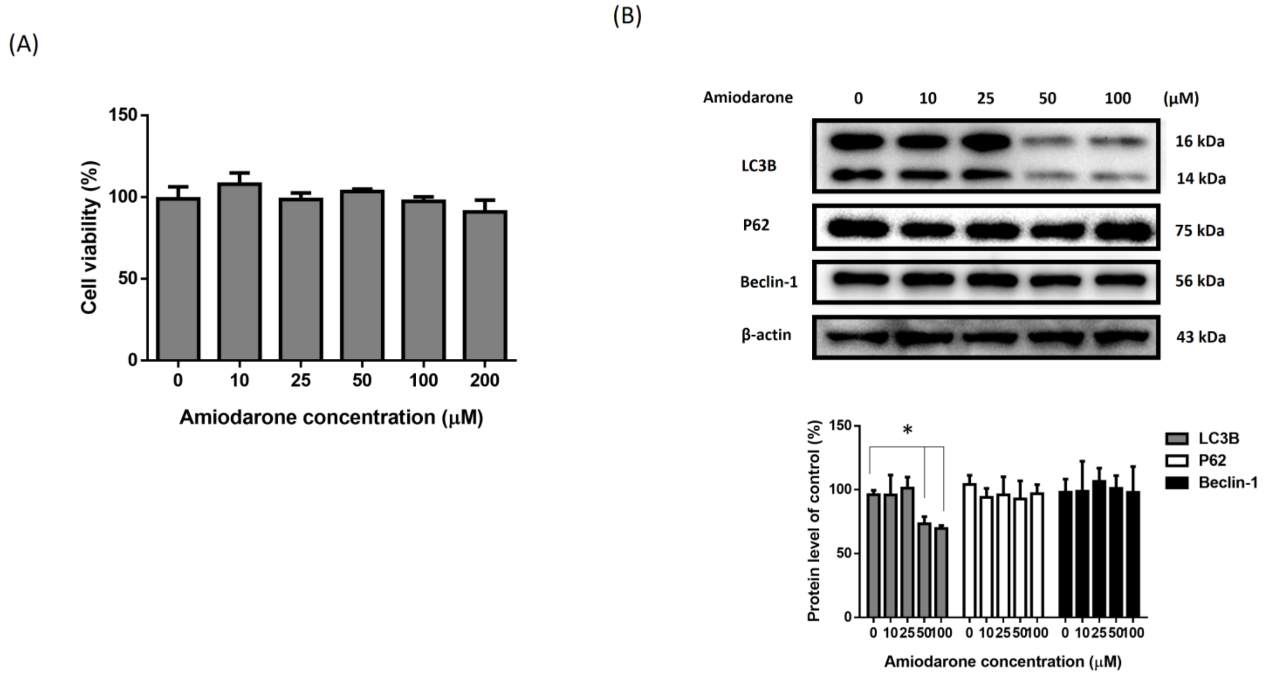

Supplementary Figure 2 Amiodarone exhibited no cytotoxicity of rat hepatocyte CRL 1439 cells without activation of autophagy. **(A)** MTT assay for cell viability of CRL 1439 cells treated with various concentrations of amiodarone for 24 hours. (B) Conversions of LC3B-I to LC3B-II and expression of P62 and beclin-1 were detected by immunoblotting following various concentrations of amiodarone treatment in CRL 1439 cells for 24 hours.
